# Supplementary material for: The Effect of Interface Diffusion on Raman Spectra of Wurtzite Short-Period GaN/AlN Superlattices
Source: Nanomaterials (Basel). 2021 Sep 14;11(9):2396. doi: 10.3390/nano11092396 (PMC8464769; doi:10.3390/nano11092396)
Supplement: Supplementary file 1 [file nanomaterials-11-02396-s001.zip › nanomaterials-1357744-supplementary.pdf]

Supplementary material
The effect of interface diffusion on Raman spectra of wurtzite short-period GaN/AlN superlattices

Valery Davydov [1,*], Eugene Roginskii [1], Yuri Kitaev [1], Alexander Smirnov [1], Ilya Eliseyev [1], Eugene Zavarin [1], Wsevolod Lundin [1], Dmitrii Nechaev [1], Valentin Jmerik [1], Mikhail Smirnov [2], Markus Pristovsek [3] and Tatiana Shubina [1]

[1] Ioffe Institute, St. Petersburg 194021, Russia; e.roginskii@mail.ioffe.ru (E.R.); yu.kitaev@mail.ioffe.ru (Y.K.); alex.smirnov@mail.ioffe.ru (A.S.); ilya.eliseyev@mail.ioffe.ru (I.E.); ezavarin@mail.ioffe.ru (E.Z.); lundin.vpegroup@mail.ioffe.ru (W.L.); nechayev@mail.ioffe.ru (D.N.); jmerik.pls@mail.ioffe.ru (V.J.); shubina@beam.ioffe.ru (T.S.)

[2] Faculty of Physics, Saint-Petersburg State University, St. Petersburg 199034, Russia; m.smirnov@spbu.ru

[3] Institute for Materials and Systems for Sustainability, Nagoya University, Nagoya 464-8601, Japan; pristovsek@imass.nagoya-u.ac.jp

* Correspondence: valery.davydov@mail.ioffe.ru; Tel.: +7-(812)-2927911

S1. The REI model

Structures of the SLs with diffuse interfaces were analyzed assuming that the cation sites in the interface regions are occupied randomly by both cations (Ga and Al) while keeping the given Ga/Al ratio constant in each atomic plane perpendicular to the *z*-axis. Thus, material with diffuse interfaces was considered as a $Ga_xAl_{1-x}N$ solid solution, in which the compositional parameter x is a linear function of the z - coordinate along the growth axis of the planar heterostructure. The idea of the REI method, which we applied to the modeling of the phonon states in GaN/AlN SLs with diffuse interfaces, is based on the concept of a "split" atom, which means that each i-th cation position can be populated by an Al atom with probability a_i or by a Ga atom with probability $1-a_i$. Thus, the number of cations in the unit cell is doubled. Correspondingly, the number of equations of the motion increased. They are solved as follows. First, we calculate all blocks of the force constant matrices for isostructural ordered GaN and AlN systems ($\mathbf{V}_{i,j}^{\text{N-N}}$, $\mathbf{V}_{i,j}^{\text{N-Al}}$, $\mathbf{V}_{i,j}^{\text{N-Ga}}$, $\mathbf{V}_{i,j}^{\text{Al-Al}}$, $\mathbf{V}_{i,j}^{\text{Ga-Ga}}$, $\mathbf{V}_{i,j}^{\text{Al-Ga}}$) and then build blocks of the REI force constant matrix $\mathbf{U}_{i,j}$ as follows:

$$\mathbf{U}_{i,j}^{\text{N-N}} = \mathbf{V}_{i,j}^{\text{N-N}},\ \mathbf{U}_{i,j}^{\text{N-Al}} = a_j\mathbf{V}_{i,j}^{\text{N-Al}}.\ \mathbf{U}_{i,j}^{\text{N-Ga}} = (1-a_j)\mathbf{V}_{i,j}^{\text{N-Ga}} \tag{1}$$

$$\mathbf{U}_{i,j}^{\text{Al-Al}} = a_ia_j\mathbf{V}_{i,j}^{\text{Al-Al}},\ \mathbf{U}_{i,j}^{\text{Ga-Ga}} = (1-a_i)(1-a_j)\mathbf{V}_{i,j}^{\text{Ga-Ga}},\ \mathbf{U}_{i,j}^{\text{Al-Ga}} = a_i(1-a_j)\mathbf{V}_{i,j}^{\text{Al-Ga}}$$

Diagonalization of the dynamic matrix based on the U force constant matrix allows determining the frequencies of the zone-center phonons in the disordered systems. Some of the solutions correspond to vibrations in which the Al and Ga atoms, located in the same position, move in phase. They describe real vibrations of a structurally disordered system. Other solutions correspond to the vibrations in which the overlapped Al and Ga atoms move in antiphase. They describe localized vibrations induced by structural

disorder. Such modes are of particular interest when one type of atom is a low-concentration impurity. A more detailed presentation of the REI model is given in Ref [S1].

In the present work, we used atom-atom potentials that were previously successfully used in modeling the lattice dynamics of both bulk GaN and AlN crystals and GaN/AlN SLs with sharp interfaces [S2]. These potentials are based on the rigid-ion approximation and include both the Coulomb long-range action and the non-Coulomb short-range action described in the framework of the Born-Karman model. The parameters $A = \frac{d^2E}{dR^2}$ and $B = \frac{1}{R}\frac{dE}{dR}$ of the Born-Karman model, taken from potential models of GaN and AlN bulk crystals, are listed in Table S1. Here E is the energy and R is the bond length in newtons per meter.

Table S1. Parameters of the Born-Karman potentials (in N/m).

Interacting atoms	A	B
Ga-N	230	−15
Al-N	240	-8
N-N	10	0
Ga-Ga	15	0
Al-Al	15	0

The cation effective charges determined from the LO-TO splitting in bulk AlN and GaN crystals are Z (Ga) = 1.14e and Z (Al) = 1.27e, where e is the electron charge. The charges of nitrogen atoms were assumed to be dependent on the cation neighbors. In wurtzite structure, each N atom is surrounded by four cations. In the SL, the N atoms are located in the center of the $NGa_{n1}Al_{n2}$ tetrahedra. The charge of each N atom is determined by the formula

$$Z(\mathrm{N}) = -\frac{1}{4}\big(n_1 Z(\mathrm{Ga}) + n_2 Z(\mathrm{Al})\big), \qquad (2)$$

Use of relation (2) allows fulfilling the electric neutrality condition.

In order to test the proposed phenomenological potential model, we calculated phonon frequencies in a bulk $Ga_xAl_{1-x}N$ solid solution in dependence on the compositional parameter x. The results are shown in Figure S1.

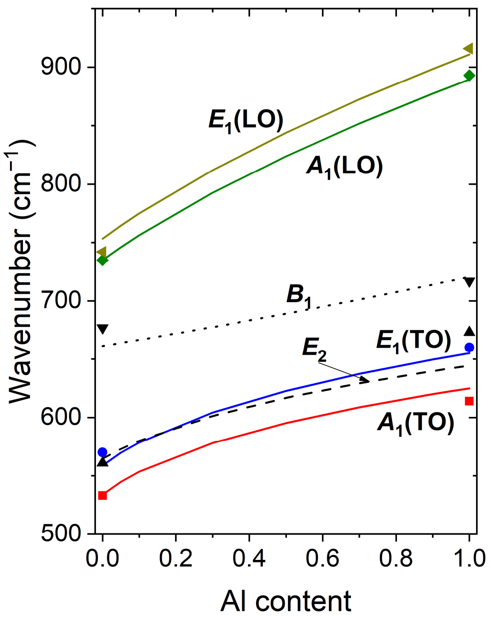

Figure S1. Frequency versus composition dependence for high-frequency phonon modes in a bulk $Ga_xAl_{1-x}N$ alloys calculated within the REI model. The symbols show the experimentally determined positions of the corresponding lines in bulk GaN and AlN.

The curves in Figure S1 agree well with the complete experimental data from Ref [S3]. This confirms our confidence in ability of the REI approach to succeed in modeling the more sophisticated spatially inhomogeneous disordered systems such as SLs.

It is noticeable that in our calculations for the bulk alloys we neglect the dependence of the structural parameters on composition. Similarly, the SL structural parameters were assumed to be independent of its total period and composition and equal to average values of the structural parameters of the bulk AlN and GaN crystals [S4]. The size of the hexagonal cell in the interface plane was taken to be 3.15 Å, and the distance along the hexagonal axis between adjacent cation layers was taken to be 2.5 Å. The use of such a fairly simple model does not allow taking into account the strain effect in the SL layers.

References

[S1] Genzel, L.; Martin, T.P.; Perry, C.H. Model for long-wavelength optical-phonon modes of mixed crystals. *Phys. Stat. Sol. (b)* **1974**, 62, 83-92 doi:10.1002/pssb.2220620108

[S2] Smirnov, M.B.; Karpov, S.V.; Davydov, V.Yu.; Smirnov, A.N.; Zavarin, E.E.; Lundin, V.V. Vibrational Spectra of AlN/GaN Superlattices: Theory and Experiment. *Phys. Solid State* **2005**, 47, 716-727. doi:10.1134/1.1913991

[S3] Grille, H.; Schnittler, Ch.; Bechsedt, F. Phonons in ternary group-III nitride alloys. *Phys. Rev. B* **2000**, 61, 6091-6105. doi:10.1103/PhysRevB.61.6091

[S4] Schulz, H.; Thieman, K.H. Crystal structure refinement of AlN and GaN. *Solid State Commun.* **1977**, 23, 815-819. doi:10.1016/0038-1098(77)90959-0
